# Immune remodeling in HPV-associated cervical disease

**DOI:** 10.3389/fcimb.2026.1918480

**Published:** 2026-09-23

**Authors:** Laura Chulenbayeva, Aidana Rakhmankulova, Nazira Kamzayeva, Samat Kozhakhmetov, Artur Kovenskiy, Madiyar Nurgaziyev, Talshyn Ukybassova, Almagul Kushugulova

**Affiliations:** 1 Laboratory of Microbiome, Center for Life Sciences, National Laboratory Astana, Nazarbayev University, Astana, Kazakhstan; 2 Department of Biomedical Sciences, School of Medicine, Nazarbayev University, Astana, Kazakhstan; 3 Clinical Academic Department of Women’s Health, Corporate Fund “University Medical Center”, Astana, Kazakhstan; 4 Department of Normal Physiology, Sechenov First Moscow State Medical University, Moscow, Russia

**Keywords:** cervical cytological grade, cervical immune microenvironment, cervicovaginal cytokines, cervicovaginal microbiome, HPV

## Abstract

**Introduction:**

Persistent high-risk HPV infection is the principal cause of cervical dysplasia, but variation in disease phenotype may also be associated with differences in the local immune and microbial environment.

**Methods:**

We characterized the cervicovaginal immune microenvironment in relation to HPV status, vaginal community state type (CST), and cytological grade.

**Results:**

HPV-positive samples showed reduced IL-4 and IL-17E/IL-25, while IFN-α2 and IL-3 varied across community state types in a non-monotonic, CST-specific manner: IFN-α2 was relatively elevated in the *Lactobacillus gasseri*-dominant CST II, and IL-3 was specifically reduced in the *Lactobacillus jensenii*-dominant CST V. Cytological category was associated with non-monotonic, marker-specific immune variation, with LSIL showing lower IL-4, IFN-γ, IL-17F, PDGF-AB/BB, IL-17E/IL-25, and PDGF-AA levels relative to NILM, while ASC-H showed a distinct rather than uniformly progressive immune profile. CLR-based multi-kingdom analyses identified a restricted set of taxon–cytokine associations after correction of the relative-abundance scale, prespecified prevalence and abundance filtering, and multiple-testing correction. Significant bacterial associations were confined to the genus level, whereas viral associations were observed at the family, genus, and species levels and fungal associations at the family level. Phocaeicola–IL-10 and Rountreeviridae–MIP-1β were the only associations that additionally remained significant after global BH-FDR correction across all taxon–immune-marker tests. Viral and fungal findings were interpreted as exploratory because of substantially lower non-bacterial sequencing depth.

**Discussion:**

These microbial associations provide additional context for the observed immune-marker differences. These findings support further investigation of multi-kingdom host–microbiome immune relationships in HPV-associated cervical abnormalities.

## 1 Introduction

Persistent infection with high-risk human papillomavirus (HR-HPV) remains the principal etiological factor in cervical intraepithelial neoplasia (CIN) and invasive cervical cancer, yet only a minority of HR-HPV infections progress to high-grade dysplasia or malignancy (Mitra et al., 2016; Kyrgiou and Moscicki, 2022). In Kazakhstan, cervical cancer remains a substantial public health burden, underscoring the need for region-specific data on the host and microbial cofactors that shape HPV-related disease trajectories in this population (Rakhmankulova et al., 2026). This discordance between viral exposure and clinical outcome has directed increasing attention toward host- and microenvironment-level cofactors that modulate the natural history of HPV infection, including local mucosal immunity and the composition of the cervicovaginal microbiome (CVM) (Mitra et al., 2020; Kyrgiou and Moscicki, 2022).

The CVM is conventionally classified into five community state types (CSTs) on the basis of dominant bacterial taxa, ranging from *Lactobacillus crispatus*-dominated communities (CST I), associated with mucosal homeostasis and low pH, to polymicrobial, *Lactobacillus*-depleted communities enriched in strict and facultative anaerobes such as *Gardnerella vaginalis*, *Fannyhessea (Atopobium) vaginae*, and *Sneathia* spp. (CST IV) (Ravel et al., 2011). CST IV and related dysbiotic states have been repeatedly and independently associated with HR-HPV persistence, accelerated progression to high-grade squamous intraepithelial lesions (HSIL), and invasive cervical cancer across diverse populations (Gardella et al., 2022; Kyrgiou and Moscicki, 2022; Tosado-Rodríguez et al., 2023). Proposed mechanisms linking dysbiosis to disease progression include loss of the protective lactic-acid barrier, biofilm formation by anaerobic taxa, activation of epithelial Toll-like receptor signaling, and induction of a chronic, low-grade mucosal inflammatory state that favors viral persistence and immune evasion (Łaniewski et al., 2018; Gardella et al., 2022).

Cervicovaginal cytokines and chemokines constitute the principal effector layer through which microbial composition is translated into local immunological consequence. Cross-sectional and longitudinal studies have shown that *Lactobacillus*-dominant CVM is generally associated with a low-inflammatory cytokine profile, whereas dysbiotic, *Lactobacillus*-depleted states correlate with elevated pro-inflammatory and chemotactic mediators, including IL-1β, IL-6, IL-8, TNF-α, MIP-1α, and MCP-1 (Łaniewski et al., 2018; Tosado-Rodríguez et al., 2023; Gonçalves-Nobre et al., 2024). Such pro-inflammatory shifts have, in turn, been implicated in promoting epithelial transformation, recruitment of myeloid and Th17 effector populations, and impaired antiviral interferon signaling across stages of cervical disease (Audirac-Chalifour et al., 2016; Łaniewski et al., 2018). However, the majority of this literature has examined the bacterial microbiome and a comparatively narrow cytokine panel in isolation, leaving the broader, multi-domain microbial ecosystem of the female genital tract spanning bacteria, viruses, and fungi and its joint relationship with the local immune milieu considerably less well characterized.

Beyond the bacterial compartment, accumulating evidence indicates that non-bacterial constituents of the CVM are not inert bystanders. The cervicovaginal virome, comprising both eukaryotic viruses and bacteriophages, shows compositional shifts that track with genital inflammation and bacterial community structure, suggesting trans-kingdom interactions within the local ecosystem (Kaelin et al., 2022). Similarly, the vaginal mycobiome though quantitatively minor relative to the bacteriome has been associated with cervical disease severity, HPV status, and CST composition, with reduced fungal diversity reported in high-grade lesions and specific taxa such as *Candida* spp. linked to local inflammatory responses that may facilitate HPV persistence (Usyk et al., 2017; Onywera et al., 2019; Godoy-Vitorino et al., 2026); in a Kazakhstani shotgun metagenomic cohort, Lactobacillus iners dominance was similarly identified as a Candida-permissive niche associated with α-9 HPV genotypes even in cytologically normal women (Kozhakhmetov et al., 2026).

Cervical cytological grade itself provides an additional, clinically anchored axis along which immune remodeling can be tracked. Cervical cytological categories provide a clinically relevant framework for examining differences in the local immune microenvironment. These categories include negative for intraepithelial lesion or malignancy (NILM), atypical squamous cells of undetermined significance (ASC-US), low-grade squamous intraepithelial lesion (LSIL), and atypical squamous cells, cannot exclude HSIL (ASC-H). These represent distinct cross-sectional cytological interpretations rather than sequential disease stages. Previous studies have reported that progression from normal cytology (NILM) to atypical squamous cells cannot exclude HSIL (ASC-H) has been associated with non-linear, module-specific changes in growth/epithelial, inflammatory, Th17/type-2, and chemokine/myeloid cytokine signatures, suggesting a transition from active immune surveillance at early stages toward immune exhaustion and dysregulation at higher grades (Audirac-Chalifour et al., 2016; Łaniewski et al., 2018). Whether and how microbial taxa across multiple kingdoms map onto this grade-dependent immunological trajectory, independent of HPV status, has not been systematically addressed.

In this study, we sought to characterize the cervicovaginal immune microenvironment in relation to HPV status, vaginal community state type, and cytological grade, and subsequently to evaluate associations between microbial taxa across the bacterial, viral, and fungal domains and the complete measured cervicovaginal immune-marker panel. By jointly profiling multi-kingdom microbiome composition and a broad cervicovaginal cytokine panel, adjusting for HPV status and lesion grade, we aimed to extend the largely bacteria-centric view of cervicovaginal dysbiosis toward a more comprehensive, ecosystem-level understanding of host–microbiome immune associations in HPV-associated cervical disease.

## 2 Materials and methods

### 2.1 Study design and participants

This cross-sectional study was conducted at a tertiary academic medical center in Astana, Kazakhstan, recruiting non-pregnant women aged 18–46 years with a regular menstrual cycle and an intact uterine cervix through the outpatient gynecology clinic of the National Research Center for Mother and Child Health, University Medical Center. Exclusions included severe chronic or immunocompromising conditions, acute inflammatory disease, recent antibiotic/probiotic/immunosuppressive use, smoking history, IUD use, prior HPV vaccination, or recent invasive gynecological procedures. Participants were included when complete clinical, cytological, HPV, immunological, and metagenomic data were available. The study was approved by the Local Bioethics Committee of the University Medical Centre (protocol No. 5, 23 May 2024) in accordance with the Declaration of Helsinki. The source metagenomic cohort comprised 311 participants. Eight participants could not be matched to available immunological data, resulting in 303 participants with integrated clinical, metagenomic, and immunological data. Seven additional participants were excluded from grade-stratified analyses because cytological grade information was unavailable, leaving a final analytical cohort of 296 participants (**Supplementary Figure S1**). Prior HPV vaccination was an exclusion criterion at recruitment; however, vaccination history for two participants became known only after enrollment and completion of data collection. These participants had already completed the study procedures and were retained in the analytical cohort as a post-enrollment protocol deviation.

### 2.2 Clinical assessment, cytology, and HPV status classification

Demographic and clinical data (age, BMI, menstrual-cycle phase) were collected via standardized questionnaires. Cervical cytology was evaluated using liquid-based preparations and classified according to the 2014 Bethesda System as negative for intraepithelial lesion or malignancy (NILM), atypical squamous cells of undetermined significance (ASC-US), low-grade squamous intraepithelial lesion (LSIL), or atypical squamous cells, cannot exclude HSIL (ASC-H). ASC-H was assigned to specimens showing squamous-cell atypia suspicious for a high-grade squamous intraepithelial lesion, including enlarged nuclei, irregular nuclear membrane contours, uneven chromatin distribution, mildly increased nuclear-to-cytoplasmic ratios, and atypical parabasal-type squamous cells. These categories were analyzed as distinct cross-sectional cytological groups and were not interpreted as sequential stages of disease progression. Histological confirmation of HSIL was not available for the ASC-H group. HPV status was determined using two complementary approaches. Targeted real-time PCR was performed using the RealBest DNA HPV High Carcinogenic Risk Genotype kit (Vector-Best, Russia), detecting HPV16, 18, 31, 33, 35, 39, 45, 51, 52, 56, 58, and 59; Ct<35 was considered positive. Shotgun metagenomic sequencing was additionally screened for HPV sequences and enabled detection of genotypes outside the targeted PCR panel. Final HPV status was assigned using a union criterion, whereby a participant was classified as HPV-positive if either PCR or metagenomic sequencing detected HPV. In the full sequenced source cohort (n=311), PCR and metagenomic sequencing showed 79.1% overall agreement: 84 samples were positive by both methods, 29 were PCR-positive/MNGS-negative, 36 were PCR-negative/MNGS-positive, and 162 were negative by both methods (Cohen’s κ=0.55). PCR-negative/MNGS-positive cases predominantly reflected HPV genotypes outside the targeted high-risk PCR panel. PCR-detected HPV types were classified as high-risk according to the carcinogenic HPV panel described above. Additional HPV genotypes detected by metagenomic sequencing were retained separately as other HPV types according to their established taxonomic and oncogenic-risk classification. In the source metagenomic cohort (n=311), 84 samples were PCR+/MNGS+, 29 were PCR+/MNGS−, 36 were PCR−/MNGS+, and 162 were PCR−/MNGS−, corresponding to 79.1% overall agreement and Cohen’s κ=0.55. Metagenomic HPV classification was based on HPV taxonomic assignments in the shotgun sequencing dataset. A separate prespecified minimum HPV read-count or genome-coverage threshold was not applied in the original analytical pipeline. Accordingly, PCR/WGS discordance is reported descriptively, and metagenomic HPV-positive cases, particularly those not confirmed by PCR, should be interpreted in the context of this limitation.

### 2.3 Cervical sample collection, DNA extraction, and sequencing

Cervical samples were collected via sterile swabs and stored at −80 °C. DNA was extracted using the ZymoBIOMICS DNA Miniprep Kit, with negative (nuclease-free water) and positive (ZymoBIOMICS Microbial Community Standard) controls. Negative extraction controls were not subjected to shotgun sequencing; therefore, prevalence-based computational contaminant identification using sequenced blanks could not be performed. This limitation was considered explicitly when curating low-abundance and biologically unexpected taxonomic assignments. DNA concentration, purity, and integrity were assessed via Qubit, NanoDrop, and Qsep400, requiring ≥1.0 μg total DNA. Whole-genome shotgun sequencing was performed on the Illumina NovaSeq 6000 (paired-end 150-bp, ~6 Gb/sample). Host reads were removed by alignment to GRCh38 using Bowtie2 v2.5.4. Taxonomic classification used Kraken2 v2.1.3 (PlusPFP database, covering bacteria, viruses, fungi, and other eukaryotes), with Bracken v3.0 refining genus/species-level abundance estimates separately for bacterial, viral, and fungal taxa. Relative kingdom-level contributions were calculated from Kraken2-assigned reads across Bacteria, Viruses, and Fungi for samples in the final analytical cohort. Effective microbial sequencing depth varied substantially among samples. In analyses of the source metagenomic dataset, the median bacterial taxonomic sequencing depth was approximately 3.84 million reads per sample (IQR 3.10–4.87 million; range 0.36–14.04 million). Non-bacterial microbial fractions were substantially lower than the bacterial fraction; in a multi-kingdom subset analysis of the same sequencing resource, median classified counts were approximately 118,233 bacterial reads, 129 fungal reads, and 26 viral reads per sample. These differences in sequencing depth and taxonomic representation were considered when interpreting low-abundance fungal and viral assignments. Because cervicovaginal specimens represent a relatively low-microbial-biomass environment and broad metagenomic reference databases may generate assignments to environmentally or anatomically implausible taxa, additional taxonomic quality control was performed before microbiome–immune association analysis. Taxa were reviewed at the full taxonomic-lineage level, and predefined animal-, plant-, soil-, marine-, rumen-, insect-, and other strongly non-cervicovaginal lineages were excluded. When an implausible genus or higher taxonomic lineage was excluded, all descendant assignments were removed. Species labels containing reference-strain or isolate designations were collapsed to the corresponding biological species where a species-level assignment was supported; unresolved strain- or isolate-level labels were not interpreted as independent species. A complete audit of excluded taxa and taxonomic consolidation is provided in **Supplementary Table 3**.

### 2.4 Community state type assignment

Cervicovaginal bacterial communities were classified into five community state types (CSTs) from species-level relative-abundance profiles. The CST-associated bacterial species with the highest relative abundance within each sample was used to define the dominant community type; no fixed numerical dominance threshold was applied. Samples in which Lactobacillus crispatus, L. gasseri, L. iners, or L. jensenii showed the highest relative abundance were classified as CST I, CST II, CST III, or CST V, respectively. Samples lacking dominance by these Lactobacillus species and characterized by predominance of Gardnerella, Fannyhessea vaginae, Prevotella, Sneathia, and/or other anaerobic taxa were assigned to CST IV. Mixed communities were therefore classified according to the taxon or community pattern with the greatest relative representation. CST was included as a categorical variable in downstream models, with CST I as the reference category. In the final analytical cohort, the CST distribution was CST I (n = 66), CST II (n = 24), CST III (n = 68), CST IV (n = 136), and CST V (n = 9).

### 2.5 Cervicovaginal cytokine, chemokine, and growth-factor profiling

Cervical swab specimens were immersed in phosphate-buffered saline (PBS) and vortexed to release cervicovaginal material from the swab. The suspensions were incubated on ice for approximately 10 min and subsequently centrifuged at approximately 4000 rpm before collection of the resulting supernatant for immune-marker analysis. The recovered cervical-swab eluate was then analyzed using the MILLIPLEX^®^ Human Cytokine/Chemokine/Growth Factor Panel A magnetic bead assay. Cervicovaginal immune mediators were quantified using the MILLIPLEX^®^ Human Cytokine/Chemokine/Growth Factor Panel A magnetic bead assay (HCYTA-60K-PX38; MilliporeSigma/Merck, Burlington, MA, USA; kit lot 4191809) on a Luminex FLEXMAP 3D^®^ platform, according to the manufacturer’s instructions. The panel comprised 37 analytes: EGF, Eotaxin, G-CSF, GM-CSF, IFN-α2, IFN-γ, IL-1α, IL-1β, IL-1RA, IL-2, IL-3, IL-4, IL-5, IL-6, IL-7, IL-8, IL-10, IL-12(p40), IL-12(p70), IL-13, IL-15, IL-17A, IL-17E/IL-25, IL-17F, IL-18, IL-22, IP-10, MCP-1, M-CSF, MIG, MIP-1α, MIP-1β, PDGF-AA, PDGF-AB/BB, TNF-α, TNF-β, and VEGF. Cervicovaginal immune mediators were quantified using the MILLIPLEX^®^ Human Cytokine/Chemokine/Growth Factor Panel A magnetic bead assay (HCYTA-60K-PX38; MilliporeSigma/Merck, Burlington, MA, USA; kit lot 4191809) on a Luminex FLEXMAP 3D^®^ platform, according to the manufacturer’s instructions. Concentrations were expressed as pg/mL. Due to limited specimen volume, samples were analyzed in single wells rather than technical duplicates. Assay performance was monitored using the manufacturer-provided Control 1 and Control 2 materials (HCYTA-6060-2; lots A6602–106 and A6602-206), with analyte-specific expected concentration ranges provided by the manufacturer. Manufacturer-reported assay precision for this panel is<15% intra-assay coefficient of variation and<20% inter-assay coefficient of variation. The instrument output additionally contained a parameter labelled “Total Events.” This parameter was generated directly by the Luminex acquisition system and was analyzed separately from individual cytokine concentrations. Because it represents an instrument/acquisition-related parameter rather than a cytokine concentration, it was not interpreted as a direct measure of cumulative immune activation.

### 2.6 Statistical analysis of immune-marker levels

Analyses were conducted in Python (pandas, NumPy, SciPy, statsmodels, matplotlib, seaborn). Concentrations were log10-transformed after adding a pseudocount of 1 to reduce right-skewness typical of cytokine concentration data; log10-transformed values approximated normal distributions more closely than raw concentrations, supporting the use of Welch’s t-test and ordinary least-squares regression on transformed values, while the non-parametric Kruskal-Wallis test was additionally used for unadjusted comparisons involving more than two groups as a distribution-free check. Unadjusted pairwise and two-group comparisons used Welch’s t-test (robust to unequal variances) on log10-transformed values, and unadjusted comparisons involving more than two groups used the Kruskal-Wallis test. Adjusted analyses were performed using ordinary least-squares regression. For HPV status, CST, and HPV type-group analyses, models included the grouping variable together with available covariates (age, BMI, and menstrual phase). For cytological-grade analyses, HPV status was additionally included as a covariate:


$$log10\left(immune-marker\;+\;1\right)\;=\;\beta �\;+\;\beta_{1}\;\times \;cytological\;grade\;+\;\beta_{2}\;\times \;age\;+\;\beta_{3}\;\times \;BMI\;+\;\beta_{4}\;\times \;menstrual\;phase\;+\;\beta_{5}\times \;HPV\;status\;+\;\epsilon$$

with HPV status added as a covariate for the cytological-grade model. Overall associations between each grouping variable and each immune marker were assessed by comparing the full adjusted model with a reduced model omitting the grouping variable using a partial F-test. Reference categories were HPV-negative, NILM, no HPV for HPV type group, and CST I for CST analyses. Benjamini-Hochberg false-discovery-rate correction was applied separately to the overall unadjusted tests, overall adjusted model-comparison tests, and regression coefficient-level tests within each grouping variable; FDR-adjusted p<0.05 was considered statistically significant. For exploratory identification of the group pairs contributing to significant overall associations, pairwise comparisons were performed using Welch’s t-tests on log10-transformed values. Pairwise p-values displayed as brackets in **Figures 1**, **2** were nominal and uncorrected and were interpreted as exploratory rather than independently multiplicity-controlled inferential tests. Pairwise p-values were additionally adjusted for multiple comparisons using the Benjamini–Hochberg false-discovery-rate procedure within each immune marker and grouping variable; both nominal p-values and FDR-adjusted q-values are displayed in **Figures 1**, **2**. As a prespecified exploratory subgroup analysis, associations between cytological grade and individual immune-marker concentrations were additionally evaluated separately according to HPV status. Among HPV-positive participants, grade-associated immune-marker differences were assessed using the same log10-transformed cytokine data and covariate-adjusted framework described above, with age and BMI included as covariates. Benjamini–Hochberg false-discovery-rate correction was applied across the tested immune markers, and markers with FDR-adjusted p<0.05 were retained for visualization in **Figure 3**. Pairwise comparisons between cytological categories were used for descriptive interpretation of significant overall associations and are shown in the figure. Among HPV-negative participants, no immune marker remained significant after FDR correction; therefore, no corresponding HPV-negative figure was generated. Because only one HPV-negative participant had ASC-H cytology, this category was not considered statistically interpretable within the HPV-negative subgroup.

**Figure 1:**
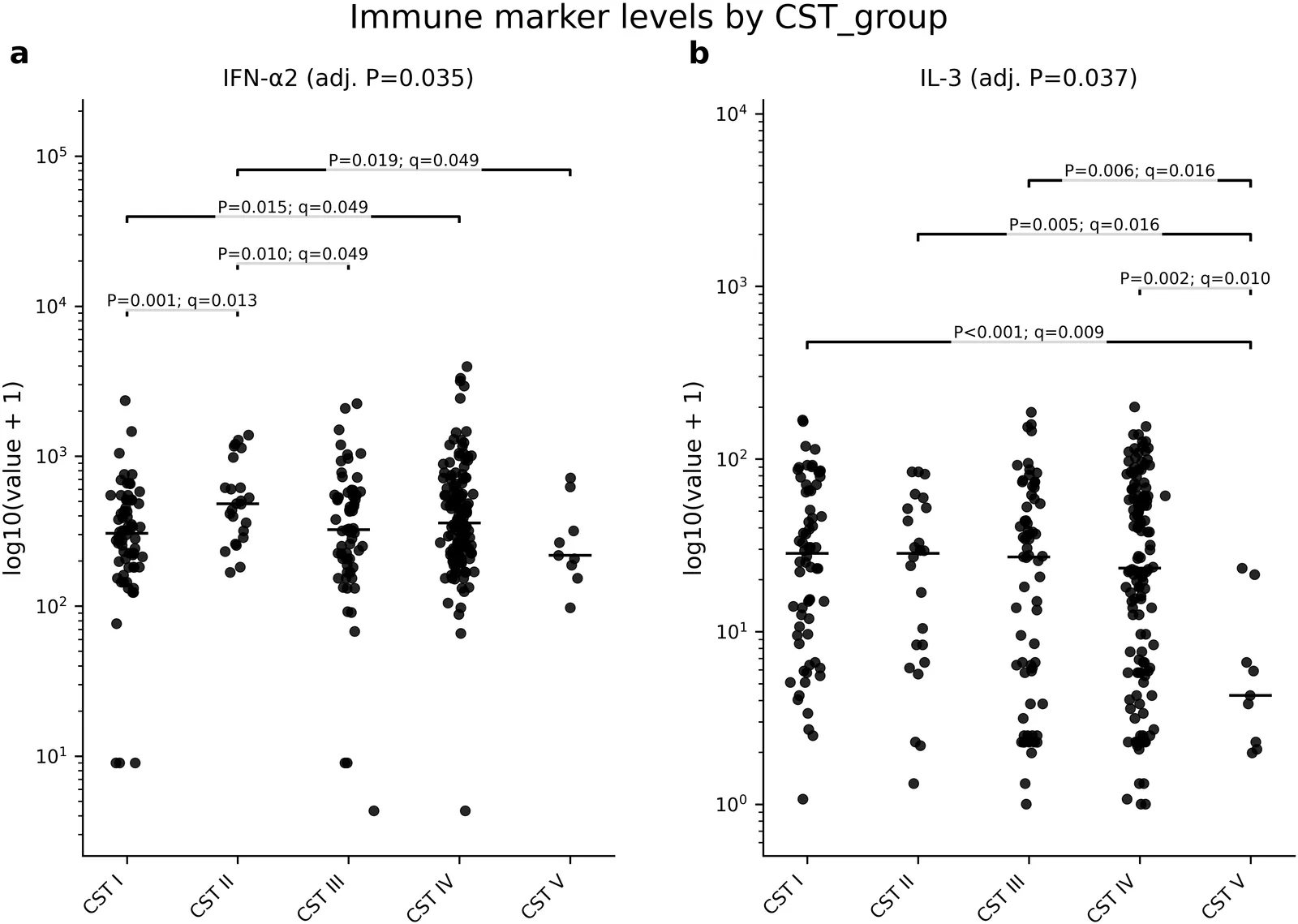
Cervicovaginal IFN-α2 and IL-3 levels differ across vaginal community state types. Cervicovaginal concentrations of **(A)** IFN-α2 and **(B)** IL-3 were compared across five bacterial community state types (CST I–V). Each point represents one participant, and horizontal bars indicate group medians. Values are shown on a log10 scale after addition of a pseudocount of 1; statistical methods are described in Section 2.6. **(A)** IFN-α2 concentrations differed significantly across CSTs (adjusted P = 0.035), with pairwise differences observed between CST I and CST II (P = 0.001), CST II and CST III (P = 0.010), CST II and CST IV (P = 0.015), and CST II and CST V (P = 0.019). **(B)** IL-3 concentrations also varied across CSTs (adjusted P = 0.037), with lower IL-3 levels in CST V compared with CST I (P<0.001), CST II (P = 0.005), CST III (P = 0.006), and CST IV (P = 0.002).

**Figure 2:**
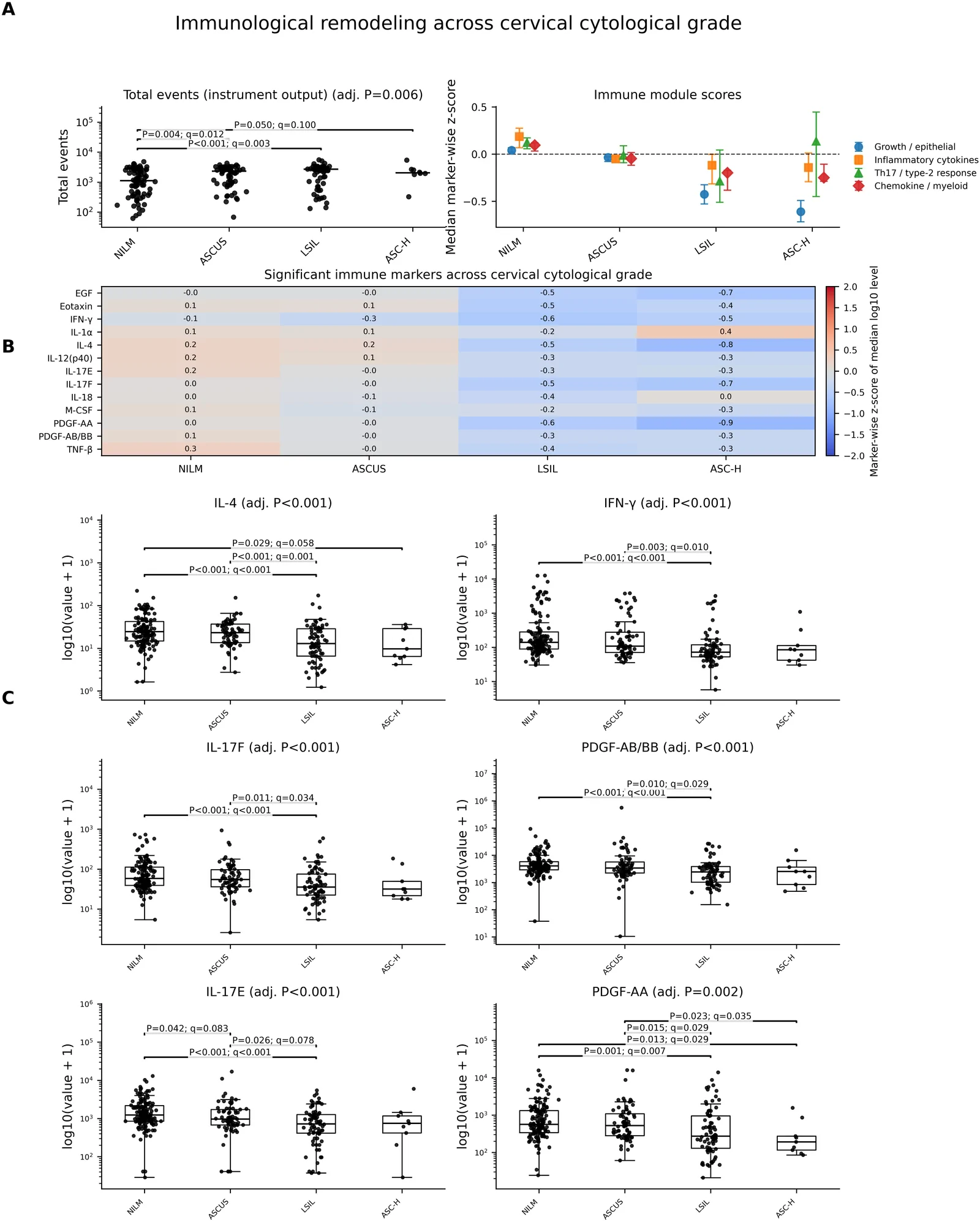
Differences in cervicovaginal immune profiles across cervical cytological categories. **(A)** Instrument-reported total events and standardized functional immune-module scores across NILM, ASC-US, LSIL, and ASC-H cytological categories. Total Events represents a parameter exported directly by the Luminex acquisition system and was evaluated as an exploratory assay/acquisition characteristic rather than as a measure of cumulative immune activation. Immune-module scores were calculated separately from marker-wise standardized cytokine concentrations. Values are displayed on a log10 scale after addition of a pseudocount of 1; statistical methods, including pairwise and overall significance testing, are described in Section 2.6. The overall association with cytological grade was evaluated using an adjusted linear regression model including age, BMI, menstrual phase, and HPV status (adjusted P = 0.006). Immune-module scores were calculated from marker-wise z-scores of log10-transformed cytokine concentrations. Lines represent median module scores within each cytological group, and error bars represent the interquartile range across markers included in each module. The dashed horizontal line indicates the cohort-wide standardized mean. **(B)** Heatmap of the 13 significant immune markers across cytological grades. Each cell represents the median marker-wise z-score of the log10-transformed concentration within a cytological group. Positive values indicate concentrations above the cohort-wide marker mean, whereas negative values indicate lower concentrations. Markers shown were selected based on significant adjusted associations with cytological grade. **(C)** Individual distributions of the six grade-associated immune markers shown in panel B: IL-4, IFN-γ, IL-17F, PDGF-AB/BB, IL-17E, and PDGF-AA. IL-17E corresponds to IL-17E/IL-25 Points represent individual participants; boxplots show the median, interquartile range, and whiskers extending to 1.5 times the interquartile range. Values are displayed as log10(value + 1). Overall adjusted p-values were derived from linear regression models including cytological grade, HPV status, age, BMI, and menstrual phase. Bracketed comparisons show nominal pairwise p-values together with the corresponding Benjamini–Hochberg FDR-adjusted q-values.

**Figure 3:**
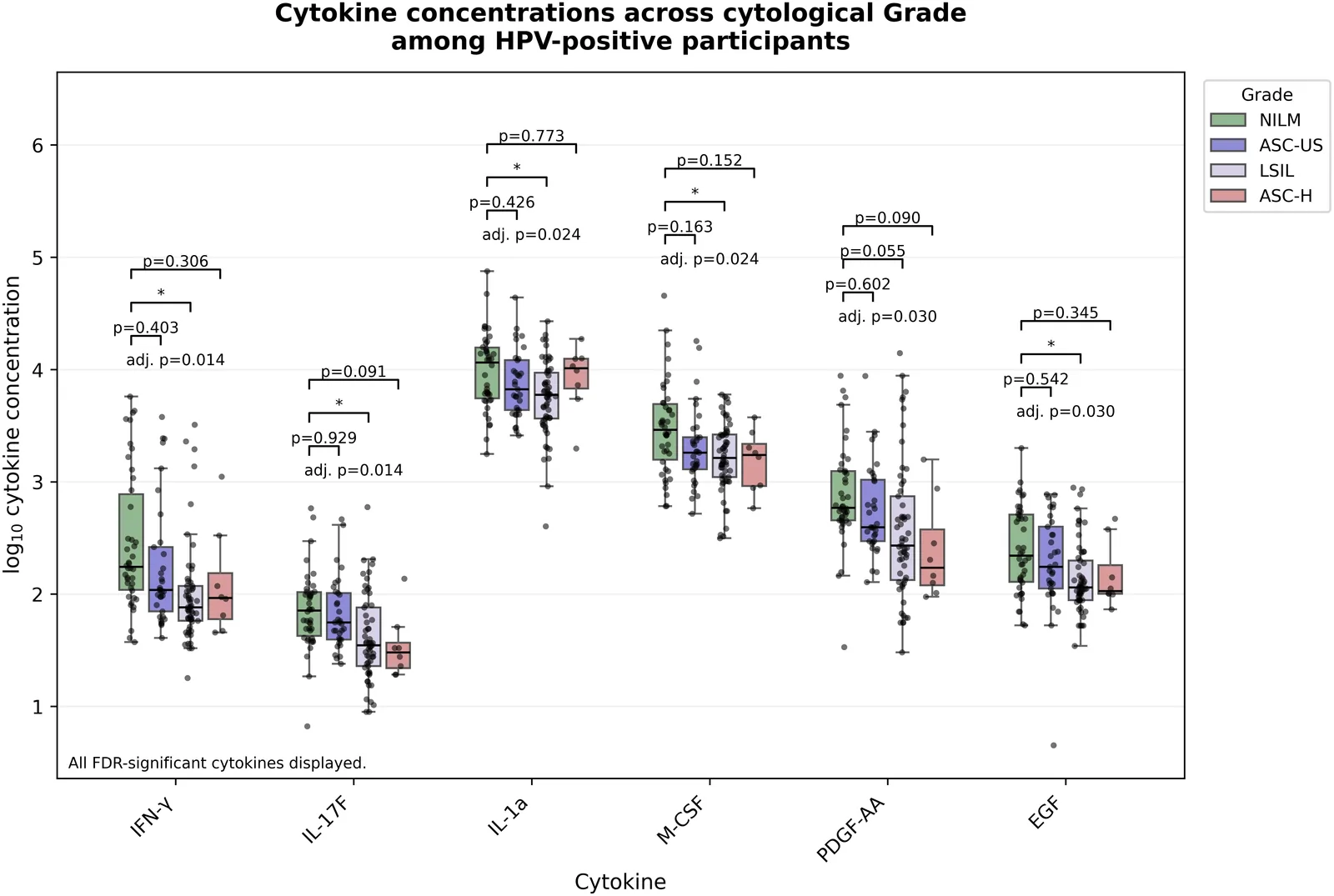
Cytokine concentrations across cytological grades among HPV-positive participants. Boxplots show log10-transformed concentrations of FDR-significant cytokines across NILM, ASC-US, LSIL, and ASC-H groups among HPV-positive participants. Individual dots represent participants; boxes indicate the interquartile range, central lines indicate medians, and whiskers indicate the distribution beyond the interquartile range. Cytokines shown were significantly associated with cytological grade in adjusted permutation-based analyses controlling for age and BMI. Brackets indicate pairwise group comparisons, with displayed values representing permutation-derived p-values. Adjusted p-values indicate Benjamini–Hochberg FDR-corrected p-values for the overall association between cytological grade and each cytokine.

### 2.7 Grade-associated immune-module analysis

Instrument-reported Total Events. The FLEXMAP 3D output contained a parameter labelled “Total Events.” This value was obtained directly from the instrument output and was not calculated as the arithmetic sum of cytokine concentrations. Total Events was evaluated as an exploratory assay/acquisition parameter across cytological groups and was not used as a composite measure of overall immune activity. Immune markers were grouped into four functional modules: growth/epithelial (EGF, VEGF, PDGF-AA, PDGF-AB/BB), inflammatory cytokines (IL-1α, IL-1β, IL-6, TNF-α, TNF-β, IL-18), Th17/type-2 (IL-4, IL-5, IL-13, IL-17A, IL-17E/IL-25, IL-17F, IL-22), and chemokine/myeloid (Eotaxin, G-CSF, GM-CSF, M-CSF, MCP-1, MIG, MIP-1α, MIP-1β, IP-10). Log10-transformed concentrations were standardized as marker-wise z-scores; module scores were the median of the grade-specific median z-scores across markers assigned to the respective module, with the interquartile range across markers displayed as error bars. These module scores were used as descriptive summaries of coordinated immune patterns and were not subjected to separate covariate-adjusted hypothesis testing. Age, BMI, and menstrual phase were selected *a priori* as covariates because of their potential biological influence on cervicovaginal immune-marker concentrations and were retained irrespective of their univariate associations with the grouping variables. HPV status was additionally included in models evaluating cytological grade because HPV prevalence differed substantially across cytological categories and therefore represented an important potential confounder of grade-associated immune-marker differences. Pregnancy history was not included in the primary multivariable models because it was available for only 124 of 296 participants; inclusion would therefore have resulted in substantial loss of observations and potentially reduced precision and representativeness.

### 2.8 Multi-kingdom microbiome-immune association analysis

Microbiome-immune associations were reanalyzed using a compositional framework. Bacterial, viral, and fungal abundance profiles were analyzed separately at the family, genus, and species levels. Sparse taxa were removed before transformation using prevalence and mean-relative-abundance criteria (family/genus: prevalence ≥5% and mean relative abundance ≥0.1%; species: prevalence ≥10% and mean relative abundance ≥0.1%). Source relative-abundance values were stored on a percentage scale and converted to proportions (0–1) before filtering; thus, 0.001 corresponds to 0.1%. Before statistical analysis, taxonomic assignments underwent additional quality control. Taxa belonging to predefined animal-, plant-, soil-, rumen-, insect-, marine- and other strongly environmental or biologically implausible lineages were removed. Exclusion was performed at the full-lineage level so that removal of an implausible higher taxon also removed its descendant species. Species names containing reference-strain or isolate identifiers were canonicalized to the corresponding species where appropriate, and duplicate species/reference-strain assignments were collapsed by summing their abundances. Unresolved strain- or isolate-level assignments were not interpreted as independent biological species. After filtering and taxonomic curation, CLR transformation was performed separately within each microbial kingdom and taxonomic level. A small pseudocount (10^-6^) was added to zero values before transformation. For each sample and retained taxon (j), the CLR-transformed abundance was defined as:


CLR(x_j) = ln(x_j) − mean[ln(x_1), …, ln(x_D)]

where (D) represents the number of retained taxa within the corresponding microbial kingdom and taxonomic level.

For each taxon–immune-marker pair, ordinary least-squares regression with HC3 heteroscedasticity-robust standard errors was fitted using log10(immune-marker concentration + 1) as the outcome and CLR-transformed taxon abundance as the predictor. Models were adjusted for HPV status and cytological grade:


$$log10\left(immune\;marker\;+\;1\right)\;=\;\beta_{0}\;+\;\beta_{1}\;\times \;CLR\;\left(taxon\right)\;+\;\beta_{2}\;\times \;HPV\;status\;+\;\beta_{3}\;\times \;cytological\;grade\;+\;\epsilon$$

All 37 measured immune mediators were included in the microbiome–immune association screen; immune markers were not selected for testing on the basis of significance in the preceding HPV-, CST-, or grade-based analyses. Associations were evaluated only when at least 20 complete observations were available after merging taxonomic, immune-marker, HPV-status, and cytological-grade data. Regression coefficients (β), robust standard errors, 95% confidence intervals, and nominal p-values were retained. Benjamini–Hochberg false-discovery-rate correction was applied separately within each prespecified microbial kingdom × taxonomic-level hypothesis family. Associations with BH-FDR-adjusted q<0.05 were retained for primary visualization. For transparency, an additional global BH-FDR correction across all 10,434 eligible adjusted taxon–immune-marker models was also calculated as a sensitivity analysis. The exact number of eligible tests included in each FDR correction family is reported in **Supplementary Table S4**. The coefficient β therefore represents the adjusted change in log10-transformed immune-marker concentration associated with a one-unit increase in the CLR abundance of the corresponding taxon relative to the geometric mean abundance of the other retained taxa within the same microbial groups and taxonomic level. For the viral component, taxonomic interpretation was additionally informed by an independent assembly-based virome workflow applied to the same source sequencing dataset. Non-host reads were assembled with MEGAHIT, viral contigs were identified using geNomad, completeness and quality were evaluated using CheckV, and viral proteins were annotated using Pharokka/PHROGs. Only viral contigs carrying viral hallmark genes were retained in the downstream virome workflow. Eukaryotic viruses and bacteriophages were interpreted separately. To evaluate whether HPV status moderated the relationship between microbial abundance and immune marker concentrations, linear models were fitted with an interaction term (taxon CLR x HPV status) adjusting for cytological grade, followed by Benjamini–Hochberg false discovery rate (FDR) correction.

### 2.9 Exploratory machine-learning analysis

Exploratory binary classifiers were constructed among HPV-positive participants to distinguish ASC-US, LSIL, and ASC-H cytology from NILM in separate analyses. The three classification datasets comprised ASC-US versus NILM (33 vs 42 participants; n=75), LSIL versus NILM (57 vs 42; n=99), and ASC-H versus NILM (8 vs 42; n=50). Because average precision depends on class prevalence, the corresponding no-skill average-precision baselines were 0.44, 0.58, and 0.16, respectively. Candidate predictors comprised the measured cervicovaginal cytokines. No outcome-based feature selection was performed. Within each training fold, cytokines were retained if at least 70% of values were non-missing and at least three unique observed values were present. Negative concentration values were treated as missing. Each retained cytokine was log10-transformed using a pseudocount equal to one-half of the smallest positive concentration observed in the corresponding training fold, and the same training-derived pseudocount was applied to the held-out data. Median imputation and z-score standardization were also fitted exclusively within the training data.

Classification was performed using L2-regularized logistic regression with balanced class weights. Model complexity was controlled through the inverse regularization parameter C, selected within an inner stratified cross-validation loop from the prespecified grid 0.01, 0.1, 1, 10, and 100. Generalization performance was estimated using repeated nested stratified cross-validation, with up to five outer folds repeated four times and an inner stratified cross-validation loop used exclusively for hyperparameter selection. Receiver operating characteristic area under the curve (AUC) and average precision (AP) were calculated from held-out outer-fold predictions. Because repeated outer cross-validation generated multiple held-out predictions per participant, predictions were averaged at the participant level before final performance estimation. 95% confidence intervals for AUC and AP were obtained by nonparametric bootstrap resampling of participants using 5,000 resamples.

Cytokine feature importance was evaluated using permutation importance applied exclusively to held-out outer test folds, with the decrease in ROC AUC after permutation used as the importance metric. Mean permutation importance and empirical 95% intervals across outer-fold estimates were reported. These models were intended solely for exploratory internal validation. Because no independent external validation cohort was available, model performance was not interpreted as clinically validated diagnostic or risk-stratification performance.

### 2.10 Data visualization

Figures were generated in Python (matplotlib, seaborn). Individual immune-marker plots showed participant-level observations and group medians on a log scale (pseudocount of 1). Heatmaps summarized standardized immune-marker patterns across grades; bubble plots summarized FDR-significant taxon-specific associations across microbial kingdoms. Bubble color represents the adjusted CLR regression coefficient (β), with red indicating positive and blue indicating negative associations, whereas bubble size represents −log10(FDR-adjusted q-value).

## 3 Results

### 3.1 Cohort characteristics relevant to immune profiling

Of the 311 participants in the source metagenomic cohort, 303 had matched immunological data. Seven of these participants lacked cytological grade information and were excluded from grade-based analyses, resulting in a final analytical cohort of 296 women stratified by cytological grade: NILM (n = 141, 47.6%), ASC-US (n = 70, 23.6%), LSIL (n = 76, 25.7%), and ASC-H (n = 9, 3.0%). Because CST V and ASC-H contained only nine participants each in the final analytical cohort, estimates involving these categories were expected to have reduced precision. Accordingly, regression coefficients and pairwise comparisons involving CST V or ASC-H are reported with 95% confidence intervals and should be interpreted cautiously as exploratory estimates rather than as stable group-specific effects. As cervical cytokine levels can be influenced by HPV status, parity, and microbiome composition, these variables were examined across grade groups prior to immunological analysis (**Supplementary Table 1**).

In the source metagenomic cohort, agreement between targeted PCR and metagenomic HPV detection was 79.1% (84 PCR+/MNGS+, 29 PCR+/MNGS−, 36 PCR−/MNGS+, and 162 PCR−/MNGS−; Cohen’s κ=0.55). PCR+/MNGS− discordance was interpreted as likely reflecting lower HPV abundance below metagenomic detection sensitivity, whereas PCR−/MNGS+ cases largely reflected detection of HPV genotypes not represented in the targeted PCR panel. By targeted PCR, the most frequently detected genotypes were HPV16 (23%), HPV52 (15%), HPV33 (12%), HPV45 (9%), HPV59 (9%), HPV39 (8%), HPV18 (8%), HPV51 (7%), HPV56 (7%), HPV31 (6%), HPV35 (6%), and HPV58 (6%). Multiple infection with two or more genotypes was identified in 42 participants by PCR.

HPV status differed markedly by grade (p< 0.001): the proportion of HPV-negative women declined from 70.2% in NILM to 11.1% in ASC-H, while HPV High Risk rose correspondingly from 12.8% to 88.9%, confirming the expected grade–HPV gradient and necessitating HPV status as a covariate in cytokine modeling. History of pregnancy also varied significantly across grades (p = 0.001), though based on a smaller subset of the cohort.

Community state type showed no significant association with cytological grade (p = 0.166), despite CST IV being numerically most frequent in ASC-H (55.6%). Age, BMI, age at menarche, menstrual cycle characteristics, parity, sexual history, contraceptive use, comorbidities, ethnicity, education, and marital status were all balanced across grade groups (all p > 0.05), arguing against these factors as major confounders of cytokine measurements in this cohort.

Overall, HPV status was the principal source of demographic heterogeneity across cytological grade groups and was accounted for accordingly in the cytokine association analyses below.

### 3.2 Determinants of the cervicovaginal immune microenvironment

To characterize the local immune environment, cytokine levels were measured in cervicovaginal swab samples.

#### 3.2.1 Immunological marker levels by HPV status

Comparison of cervicovaginal cytokine levels between HPV-negative and HPV-positive patients revealed significant differences in two key immune markers (**Figure 4**). IL-4 levels were significantly higher in HPV-negative compared to HPV-positive samples (adjusted P<0.001), with median levels approximately 25 pg/mL versus 15 pg/mL, respectively, suggesting suppression of Th2-associated immune responses upon HPV infection. Similarly, IL-17E/IL-25 levels were significantly higher in HPV-negative individuals (adjusted P = 0.002). These findings demonstrate cross-sectional differences in Th2-associated and epithelial-derived immune mediators according to HPV status.

**Figure 4:**
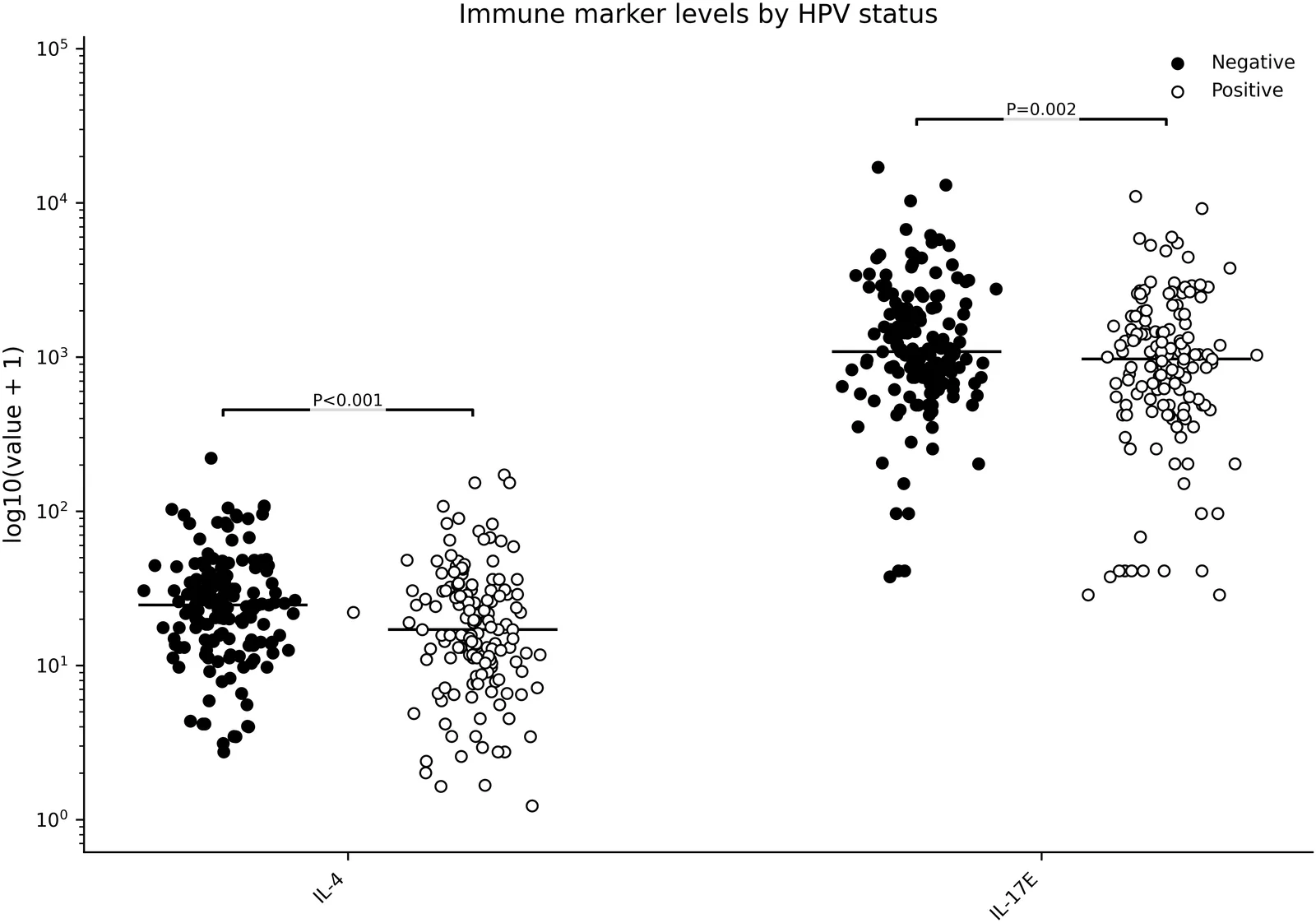
Reduced cervicovaginal IL-4 and IL-17E levels in HPV-positive participants. Cervicovaginal concentrations of IL-4 and IL-17E were compared between HPV-negative and HPV-positive participants. Each point represents one participant; filled circles indicate HPV-negative samples and open circles indicate HPV-positive samples. Horizontal bars represent group medians. Values are displayed on a log10 scale after addition of a pseudocount of 1. Overall adjusted p-values were obtained from linear regression models adjusted for age, BMI, and menstrual phase. IL-4 concentrations were significantly lower in HPV-positive participants than in HPV-negative participants (adjusted P<0.001), as were IL-17E concentrations (adjusted P = 0.002).

#### 3.2.2 Immunological marker levels by community state type

Analysis of cervicovaginal cytokine levels across community state types (CST I-V) revealed significant differences in antiviral and hematopoietic immune markers (**Figure 1**).

IFN-α2 levels differed significantly across CSTs (**Figure 1A**, adjusted P = 0.035), with the pattern driven primarily by relatively elevated levels in CST II rather than by a monotonic decline across CSTs. Significantly higher IFN-α2 concentrations were observed in CST II compared to CST I (P = 0.001, q = 0.013), CST III (P = 0.010, q = 0.049), and CST V (P = 0.019, q = 0.049). An additional difference was observed between CST I and CST IV (P = 0.015, q = 0.049), with CST V exhibiting the lowest median values (~250 pg/mL). Because the highest IFN-α2 levels occurred in CST II and the lowest in CST V - both Lactobacillus-dominant states - this pattern does not support a straightforward, monotonic suppression of type I interferon with increasing community diversity; rather, it reflects CST-specific differences whose biological basis remains to be established.

IL-3 levels similarly varied significantly across CSTs (**Figure 1B**). CST I and CST II maintained comparable median levels (~25–30 pg/mL), whereas CST V demonstrated markedly reduced IL-3 concentrations relative to CST I (P<0.001, q = 0.009), CST II (P = 0.005, q = 0.016), CST III (P = 0.006, q = 0.016) and CST IV (P = 0.002, q = 0.010). Because CST V represents a *Lactobacillus jensenii*–dominant community rather than a Lactobacillus-depleted, polymicrobial community (CST IV), this reduction in IL-3 - a hematopoietic, myeloid-supportive cytokine - is not attributable to the anaerobe-dominated community type and instead identifies a CST-V-specific signal.

Collectively, these findings indicate that cervicovaginal IFN-α2 and IL-3 differ across community state types in a CST-specific manner, with relative elevation of IFN-α2 in CST II and a specific reduction of IL-3 in CST V.

#### 3.2.3 Differences in cervicovaginal immune profiles across cytological categories

The instrument-reported Total Events parameter differed across cytological categories (adjusted P = 0.006), with higher values observed in ASC-US and LSIL compared with NILM. Because Total Events represents an acquisition-related parameter rather than a cytokine concentration measure, this finding was considered exploratory and was not interpreted as evidence of increased overall immune activation. In contrast, the standardized immune-module scores summarized coordinated patterns across biologically related cytokine and chemokine profiles across cytological grades revealed significant grade-dependent alterations in 13 immune mediators spanning all four functional modules, with all analyses adjusted for age, BMI, menstrual phase, and HPV status. Median scores for all four functional immune modules Growth/epithelial, Inflammatory cytokines, Th17/type-2 response, and Chemokine/myeloid were positive at NILM and lower values in ASCUS, LSIL and ASC-H. The Growth/epithelial module showed the most pronounced and sustained decline, while the Th17/type-2 module showed a distinct ASC-H profile relative to LSIL (**Figure 2A**).

Profiling of the significant immune markers showed a consistent pattern across grades: values were near zero or slightly positive in NILM and ASCUS, were negative for all markers except IL-1α and IL-18 in LSIL, and in ASC-H were predominantly more negative than in LSIL, with the exception of IL-1α and IL-18 (**Figure 2B**). The lowest values at ASC-H were observed for PDGF-AA (z-score −0.9), IL-4 (−0.8), EGF and IL-17F (−0.7 each). IL-1α was the only marker with a positive z-score at ASC-H (+0.4), following a negative value at LSIL (−0.2); IL-18 returned to 0.0 at ASC-H after −0.4 at LSIL. These findings further illustrate that the grade-associated immune profile was marker-specific and non-monotonic rather than a uniform progressive decrease across cytological categories.

Individual distributions of six representative grade-associated immune markers are shown in **Figure 2C**: IL-4, IFN-γ, IL-17F, PDGF-AB/BB, IL-17E, and PDGF-AA. Overall adjusted associations with cytological category were significant for IL-4 (P< 0.001), IFN-γ (P< 0.001), IL-17F (P< 0.001), PDGF-AB/BB (P< 0.001), IL-17E (P< 0.001), and PDGF-AA (P = 0.002). These markers generally showed lower concentrations in LSIL and/or ASC-H than in NILM or ASCUS, although the magnitude and pairwise pattern differed among individual mediators. Collectively, these results demonstrate that cervicovaginal immune-marker profiles differ across cytological categories, with several mediators showing lower concentrations in LSIL and ASC-H that in NILLM or ASC-US. Because the study is cross-sectional and cytological categories do not represent longitudinal stages within individual participants, these patterns should not be interpreted as evidence of temporal immune progression or exhaustion.

#### 3.2.4 HPV-stratified immune-marker differences across cytological grade

Among HPV-negative participants, none of the cytokines reached FDR-significance for association with cytological grade. The ten cytokines with the strongest (unadjusted) pairwise signal — IL-17E, Eotaxin, IL-17F, MIG, IL-1RA, G-CSF, PDGF-AA, IL-15, MCP-1, and IFN-α2 — showed nominal pairwise p-values ranging from 0.133 to 0.942 and adjusted p-values from 0.236 to 0.754, with no consistent direction of change across NILM, ASC-US, and LSIL grades. ASC-H was represented by a single participant in this HPV-negative subgroup, precluding statistical comparison for that grade. Among HPV-positive participants, six cytokines remained significantly associated with cytological grade after FDR correction: IFN-γ, IL-17F, IL-1α, M-CSF, PDGF-AA, and EGF (**Figure 3**).

#### 3.2.5 Exploratory machine-learning discrimination of cytological grade

Among HPV-positive participants, exploratory cytokine-based classifiers were further evaluated using repeated nested stratified cross-validation with L2-regularized logistic regression (**Figure 5**). The ASC-US-versus-NILM model showed essentially chance-level discrimination, with an AUC of 0.51 (95% CI, 0.38–0.64) and average precision of 0.44 (95% CI, 0.33–0.62), which was approximately equal to the no-skill average precision (AP) baseline of 0.44.

**Figure 5:**
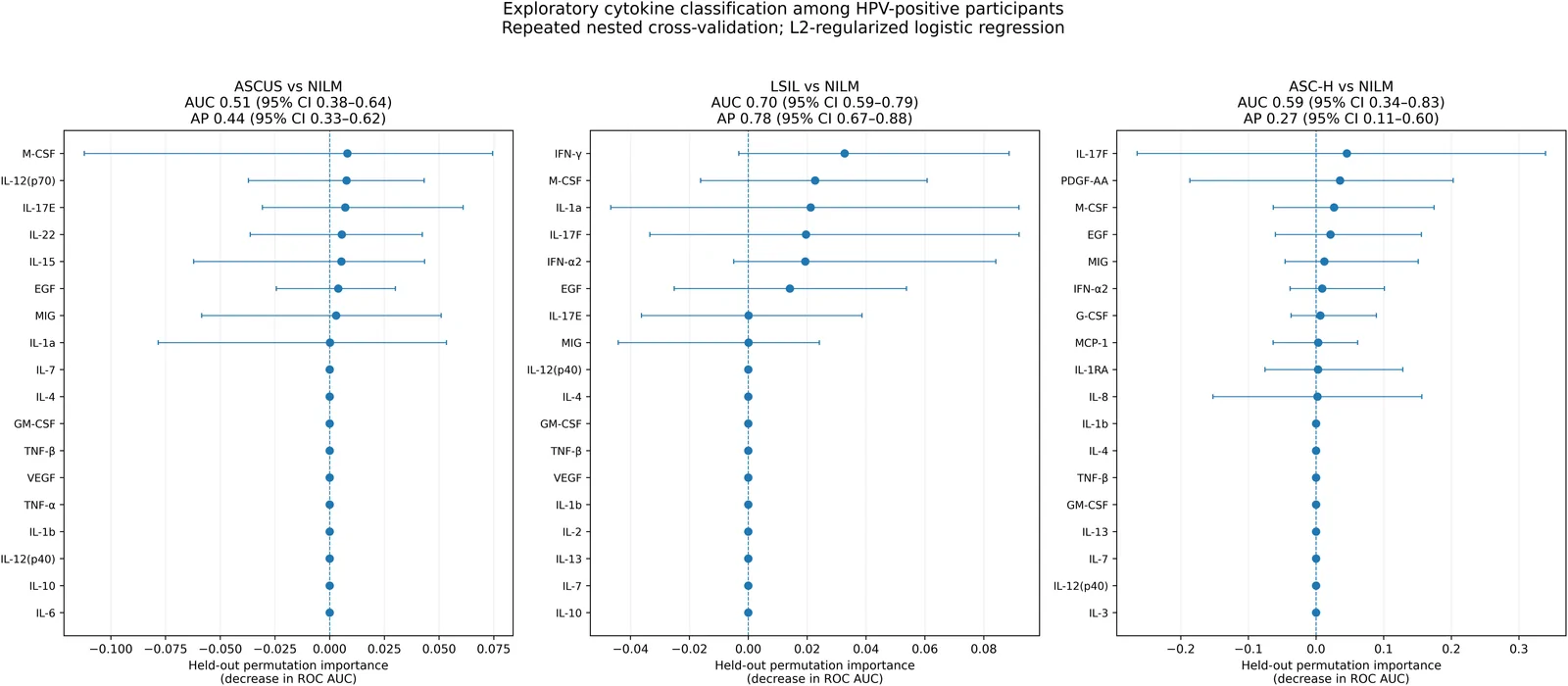
Exploratory cytokine classification of cytological categories among HPV-positive participants. L2-regularized logistic-regression models were evaluated using repeated nested stratified cross-validation for ASCUS versus NILM, LSIL versus NILM, and ASC-H versus NILM. Points indicate mean held-out permutation importance, expressed as the decrease in ROC AUC after feature permutation, and horizontal bars indicate empirical 95% intervals across outer test-fold estimates. Model discrimination is reported as participant-level AUC and average precision (AP) with 95% bootstrap confidence intervals. The LSIL-versus-NILM model showed the strongest internal discrimination (AUC 0.70, 95% CI 0.59–0.79; AP 0.78, 95% CI 0.67–0.88), whereas ASCUS-versus-NILM and ASC-H-versus-NILM showed limited discrimination. All analyses were exploratory and internally validated only.

The LSIL-versus-NILM comparison showed the strongest internal discrimination, with an AUC of 0.70 (95% CI, 0.59–0.79) and average precision of 0.78 (95% CI, 0.67–0.88), compared with a no-skill AP baseline of 0.58. Held-out permutation importance showed the largest positive mean contributions for IFN-γ, M-CSF, IL-1α, IL-17F, IFN-α2, and EGF; however, the 95% intervals for individual feature-importance estimates crossed zero, indicating substantial uncertainty in the ranking of individual cytokines.

For ASC-H versus NILM, the model achieved an AUC of 0.59 (95% CI, 0.34–0.83) and average precision of 0.27 (95% CI, 0.11–0.60), compared with a no-skill AP baseline of 0.16. Although IL-17F and PDGF-AA had the largest positive mean permutation-importance estimates, their intervals were broad and crossed zero. Given the small number of HPV-positive ASC-H participants (n=8), this comparison was considered highly uncertain and hypothesis-generating.

Overall, only the LSIL-versus-NILM comparison demonstrated moderate internal discriminatory performance. None of the models underwent external validation, and the analyses were therefore interpreted as exploratory rather than as clinically applicable diagnostic or risk-stratification models.

### 3.3 Multi-kingdom microbiome–immune associations

To characterize associations between microbial community composition and the local immune environment, CLR-transformed bacterial, viral, and fungal abundances were evaluated against all 37 measured cervicovaginal immune mediators using models adjusted for HPV status and cytological grade. Taxa were filtered using the prespecified prevalence thresholds before CLR transformation, and Benjamini–Hochberg correction was applied separately within each microbial kingdom × taxonomic-level hypothesis family. Associations with FDR-adjusted q<0.05 are shown in **Figures 6**–**8**, while the complete set of eligible models is reported in **Supplementary Table S4**.

**Figure 6:**
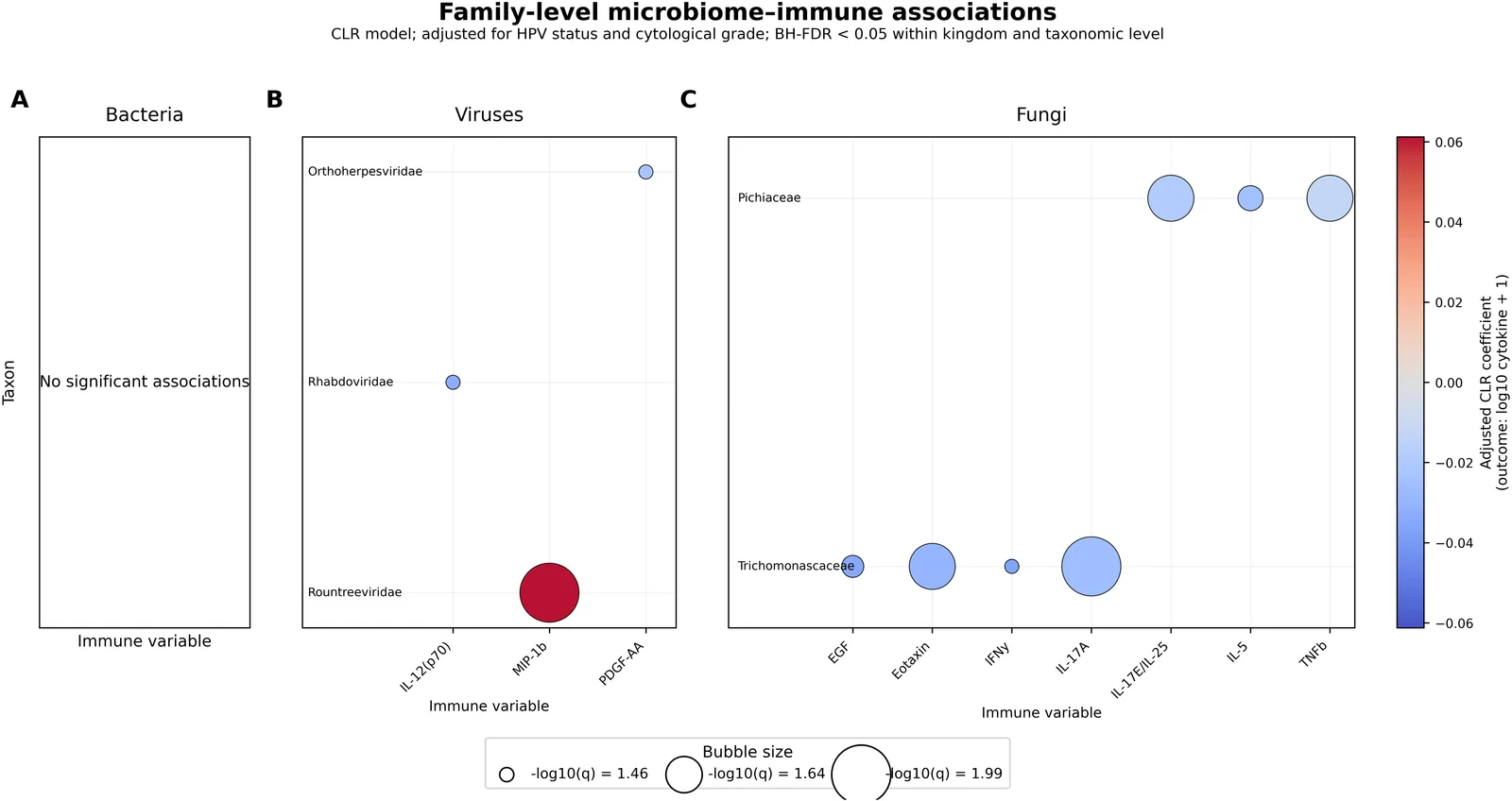
Family-level microbiome–immune associations. CLR-transformed bacterial, viral, and fungal family abundances were evaluated in multivariable linear regression models adjusted for HPV status and cytological grade. Panels show **(A)** bacteria, **(B)** viruses, and **(C)** fungi. Only associations meeting Benjamini–Hochberg FDR q<0.05 within each kingdom × taxonomic-level hypothesis family are displayed. No bacterial family-level association remained significant after FDR correction. Bubble color represents the adjusted CLR regression coefficient (β), with red indicating positive and blue indicating negative associations; bubble size represents −log10(FDR-adjusted q-value). The immune-marker outcome was log10-transformed after addition of 1.

**Figure 7:**
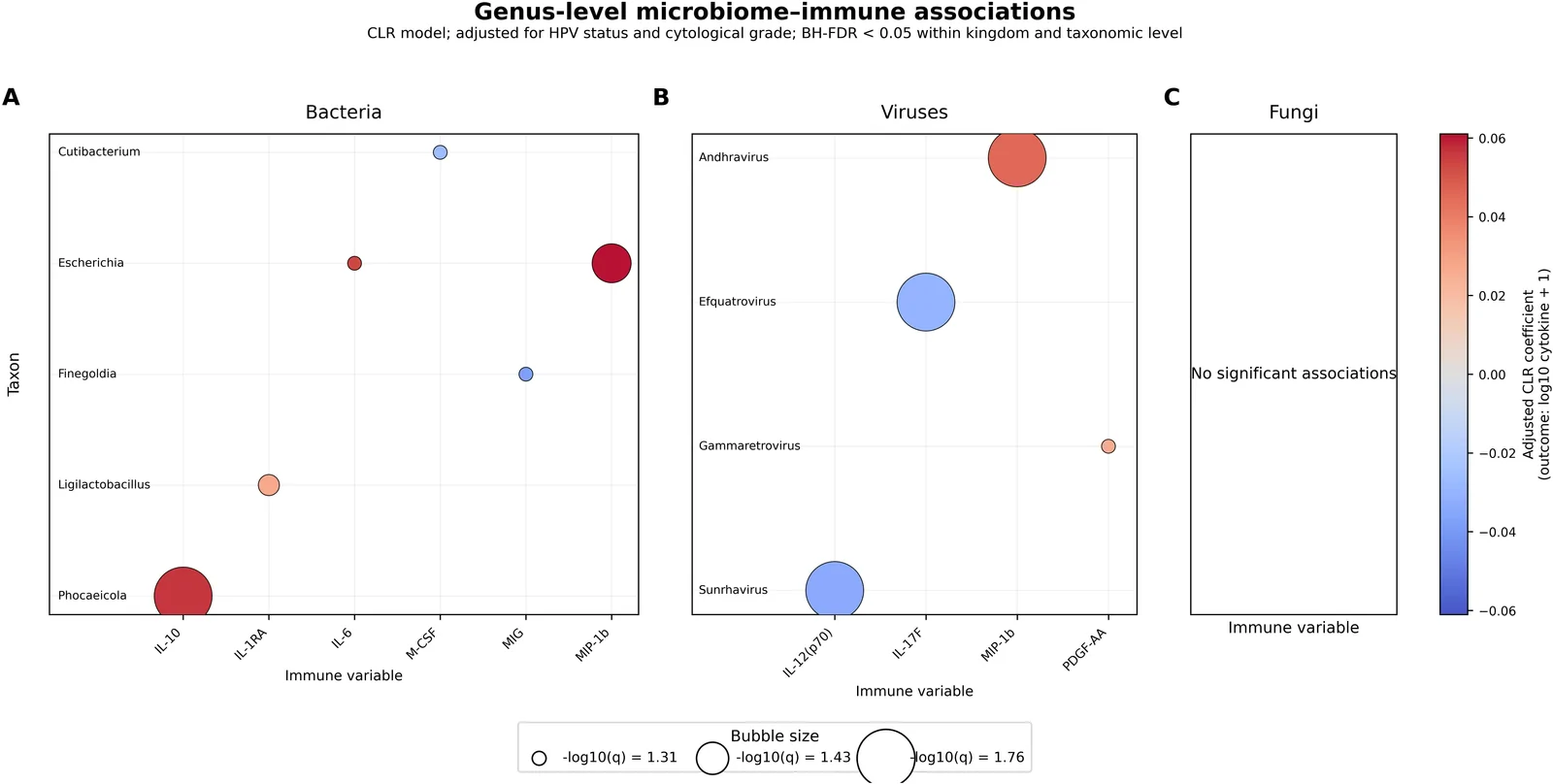
Genus-level microbiome–immune associations. CLR-transformed bacterial, viral, and fungal genus abundances were evaluated in multivariable linear regression models adjusted for HPV status and cytological grade. Panels show **(A)** bacteria, **(B)** viruses, and **(C)** fungi. Only associations meeting Benjamini–Hochberg FDR q<0.05 within each kingdom × taxonomic-level hypothesis family are displayed. No fungal genus-level associations remained significant after FDR correction. Bubble color represents the adjusted CLR regression coefficient (β), and bubble size represents −log10(FDR-adjusted q-value).

**Figure 8:**
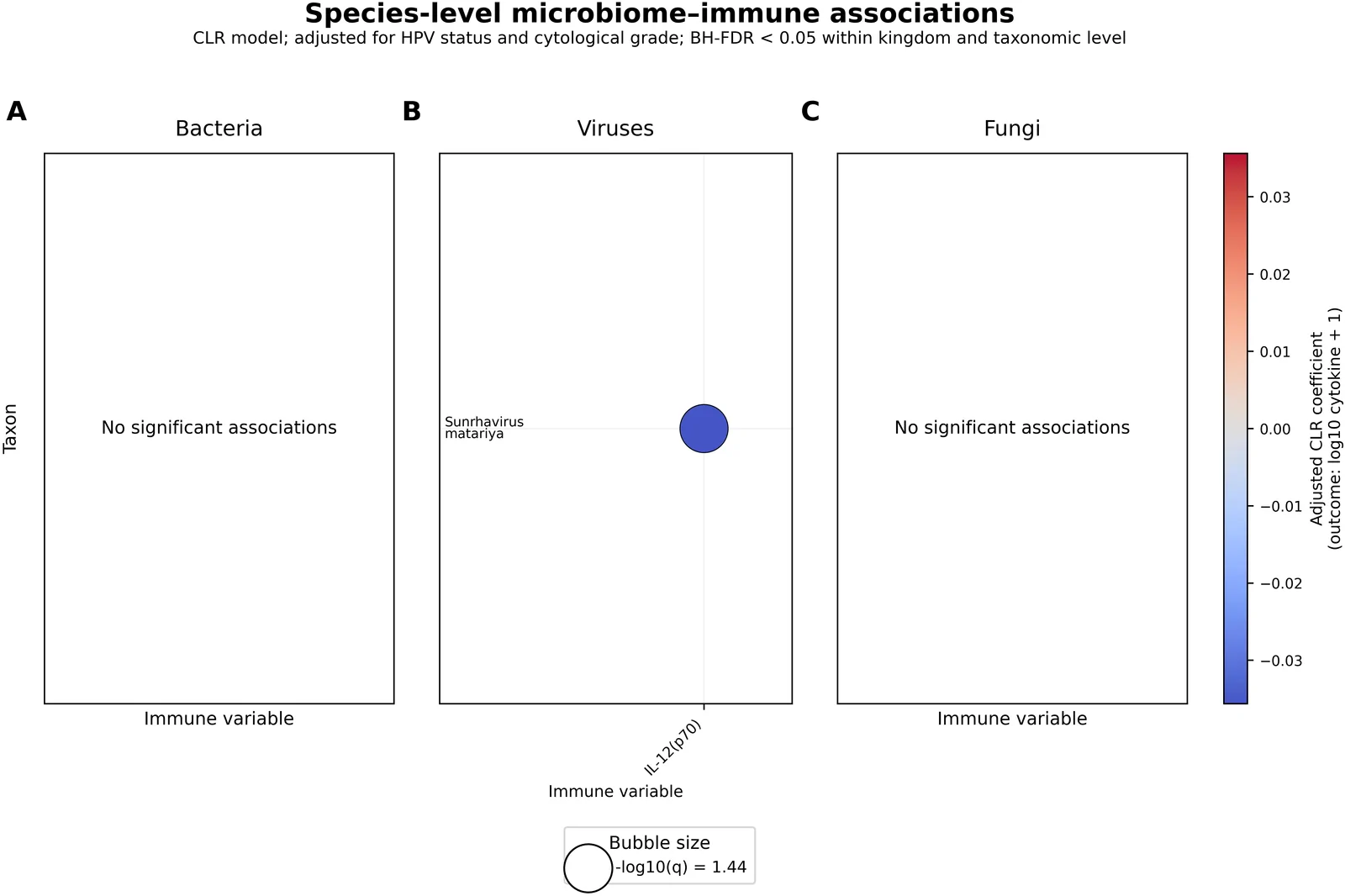
Species-level microbiome–immune associations. CLR-transformed bacterial, viral, and fungal species abundances were evaluated in multivariable linear regression models adjusted for HPV status and cytological grade. Panels show **(A)** bacteria, **(B)** viruses, and **(C)** fungi. Only associations meeting Benjamini–Hochberg FDR q<0.05 within each kingdom × taxonomic-level hypothesis family are displayed. No bacterial or fungal species-level associations remained significant after FDR correction. Bubble color represents the adjusted CLR regression coefficient (β), and bubble size represents −log10(FDR-adjusted q-value). Viral species-level associations are considered exploratory because of the low viral sequencing depth and absence of independent taxon-specific validation.

At the family level (**Figure 6**), no bacterial associations remained significant after FDR correction. Among viral families, Rountreeviridae was positively associated with MIP-1β, whereas Orthoherpesviridae was negatively associated with PDGF-AA and Rhabdoviridae was negatively associated with IL-12(p70). Among fungi, Trichomonascaceae showed inverse associations with IL-17A, Eotaxin, EGF, and IFN-γ. Pichiaceae was inversely associated with IL-17E/IL-25, TNF-β, and IL-5. At the genus level (**Figure 7**), six bacterial associations remained significant. Phocaeicola was positively associated with IL-10; Escherichia was positively associated with MIP-1β and IL-6; Ligilactobacillus was positively associated with IL-1RA; Cutibacterium was negatively associated with M-CSF; and Finegoldia was negatively associated with MIG. Viral genus-level associations included a negative association between Efquatrovirus and IL-17F, a positive association between Andhravirus and MIP-1β, a negative association between Sunrhavirus and IL-12(p70), and a positive association between Gammaretrovirus and PDGF-AA. No fungal genus-level associations remained significant after FDR correction. At the species level (**Figure 8**), no bacterial or fungal associations remained significant after FDR correction. The only significant viral species-level association was an inverse association between Sunrhavirus matariya and IL-12(p70). Across all nine hypothesis families, 21 associations met the prespecified within-kingdom × taxonomic-level BH-FDR threshold. When a more conservative global BH-FDR correction was additionally applied across all 10,434 adjusted taxon–immune-marker tests, only two associations remained significant: Phocaeicola with IL-10 and Rountreeviridae with MIP-1β. Thus, most of the associations shown in **Figures 6**-**8** should be interpreted as hypothesis-family-specific signals rather than associations robust to correction across the complete multi-kingdom test set.

While HPV status was controlled for in the primary models (**Figures 6**-**8**), formal testing revealed no significant moderation/interaction effects by HPV status across the microbiome-immune associations. This absence of significant interaction should not be interpreted as evidence that the associations are independent of HPV status, particularly given the limited statistical power of interaction testing.

Overall, the compositional analysis identified a sparse and taxonomic-level-specific pattern of microbiome–immune associations rather than a broad convergent cytokine signature. Bacterial signals were restricted to the genus level, fungal signals to the family level, and viral signals were detected across family, genus, and species levels. Given the substantially lower viral and fungal sequencing depth and the absence of sequenced extraction-negative controls, non-bacterial associations were considered exploratory. Complete results, including nonsignificant associations, regression coefficients, robust standard errors, confidence intervals, nominal p-values, within-family q-values, and global q-values, are provided in **Supplementary Table S4**.

## 4 Discussion

This study provides an integrated, multi-kingdom characterization of the cervicovaginal immune microenvironment in relation to HPV status, vaginal community state type, cytological grade, and microbiome composition spanning bacteria, viruses, and fungi. HPV infection and CST group were each associated with attenuation of innate antiviral and Th2-associated signaling. Immune-marker patterns differed non-monotonically across cytological categories, rather than showing a simple ordered gradient from NILM through ASC-H. Microbiome–immune associations were observed across bacterial, viral, and fungal domains, with distinct immune-marker patterns according to microbial kingdom and taxonomic level. The lower IL-4 and IL-17E/IL-25 concentrations observed in HPV-positive participants are consistent with previously reported differences in local Th2-associated and epithelial immune signaling in HPV infection. Experimental studies have described HPV immune-evasion mechanisms, but the present cross-sectional analysis cannot determine whether HPV directly caused the observed cytokine differences (Stanley, 2010; Łaniewski et al., 2018). Even though HPV has established immune-evasion mechanisms in experimental systems, the cross-sectional associations observed in this work cannot determine whether HPV directly caused the lower cytokine levels. The CST-specific differences observed here - relative elevation of IFN-α2 in CST II and reduction of IL-3 in CST V - do not follow a simple monotonic pattern across CST groups. Notably, the lowest IL-3 levels occurred in CST V, a *Lactobacillus jensenii*-dominant rather than *Lactobacillus*-depleted community, so these findings cannot be straightforwardly attributed to the polymicrobial CST IV state. They are nonetheless broadly compatible with reports that vaginal community composition modulates local antiviral and myeloid-supportive signaling (Gardella et al., 2022; Tosado-Rodríguez et al., 2023), although the CST-specificity and direction observed here warrant cautious interpretation and replication. Mechanistically, depletion of lactic-acid-producing *Lactobacillus* spp. removes a key suppressor of bacterial biofilm formation and epithelial Toll-like receptor activation — a systems-level interaction recently formalized in a triad model linking HPV, the cervicovaginal microbiome, and bacterial biofilm dynamics (Nazarova et al., 2026) — a process exemplified by *Fannyhessea vaginae*, which has been shown to activate TLR2/NF-κB signaling in cervicovaginal epithelial cells and drive pro-inflammatory cytokine output (Łaniewski et al., 2018; Gardella et al., 2022). Reduced type I interferon tone combined with an anaerobe-enriched bacterial community could create a local environment less capable of clearing incident HPV infection, consistent with longitudinal evidence linking taxa such as *Gardnerella* to impaired HR-HPV clearance and accelerated progression to precancer (Mitra et al., 2020).

The grade-dependent immune remodeling observed here a rise in total events alongside a biphasic decline-and-partial-recovery pattern across individual cytokine modules extends previous reports of non-linear immune trajectories in cervical carcinogenesis (Audirac-Chalifour et al., 2016; Łaniewski et al., 2018). The distinct profiles of IL-18, IL-1α, MIP-1β, and Eotaxin across cytological grades reveal non-linear patterns, characterized by lower relative levels in LSIL samples compared to higher levels observed in NILM/ASC-US and partial re-elevation of IL-1α in ASC-H samples. Rather than a uniform continuum, these cross-sectional differences suggest that different cytological categories exhibit distinct local immune microenvironments, with LSIL marked by lower baseline chemokine levels relative to both low-grade atypia (ASC-US) and high-grade lesion categories (ASC-H). Although all ASC-H specimens in this cohort demonstrated cytomorphological abnormalities suspicious for high-grade squamous intraepithelial disease, including nuclear enlargement, irregular nuclear contours, abnormal chromatin distribution, and atypical parabasal-type cells, histological confirmation was not available. This pattern parallels the broader concept, established in invasive cervical cancer, that advanced cervical neoplasia is accompanied by elevated TNF-α, TNF-β, MIP-1α, GM-CSF, and IL-10, suggestive of a shift toward an immunosuppressive, tumor-permissive cytokine milieu (Łaniewski et al., 2018). The lower growth/epithelial module scores observed in LSIL and ASC-H may indicate differences in epithelial-associated signaling across cytological categories; however, epithelial barrier integrity was not directly measured in this study.

The exploratory machine-learning analysis provided a multivariable assessment of whether cervicovaginal cytokine profiles contained discriminatory information beyond individual marker comparisons. After repeated nested cross-validation and regularization, the LSIL-versus-NILM classifier showed moderate internal discrimination (AUC 0.70, 95% CI 0.59–0.79), whereas ASCUS-versus-NILM discrimination was essentially at chance level (AUC 0.51, 95% CI 0.38–0.64). Performance for ASC-H versus NILM was also limited and highly uncertain (AUC 0.59, 95% CI 0.34–0.83), reflecting the very small ASC-H subgroup. Although several cytokines, including IFN-γ, M-CSF, IL-1α, IL-17F, IFN-α2, and EGF, showed positive mean permutation importance in the LSIL comparison, confidence intervals for individual feature-importance estimates crossed zero. Consequently, these variables should not be interpreted as stable predictive biomarkers. Rather, the classification results provide hypothesis-generating evidence that multivariate cytokine patterns may distinguish HPV-positive LSIL from NILM within this cohort, a finding that requires replication and external validation before any clinical interpretation. Notably, no cytokine reached FDR-significance for cytological grade among HPV-negative participants, indicating that grade-associated cytokine difference in this cohort is largely restricted to, or at least substantially amplified within, the context of HPV infection. Taken together, these classification analyses suggest that cytokine-based discrimination of cytological grade showed its highest internally cross-validated discrimination for the comparison between HPV-positive NILM and LSIL participants and is constrained both by sample size at the small ASC-H subgroup and by the absence of detectable cytokine remodeling in the HPV-negative population.

The restricted, taxon-specific pattern observed in our study is consistent with previous cervicovaginal studies showing that microbial community composition is linked to selective, rather than uniform, changes in local immune mediators. Łaniewski et al. demonstrated that cervicovaginal microbiota composition was associated with distinct immune signatures across HPV infection and cervical carcinogenesis (Łaniewski et al., 2018). while Tosado-Rodríguez et al. found that greater cervicovaginal microbial diversity and dysbiosis were associated with inflammatory and trafficking-related cytokines, including IL-6, MIP-1α, MCP-1, IP-10, and anti-inflammatory IL-10 (Tosado-Rodríguez et al., 2023). More recently, Yonezawa et al. showed that cervicovaginal bacterial community patterns and the abundance of Gardnerella spp. and Fannyhessea vaginae were associated with variation in IL-1β, IL-6, CXCL8, IL-10, and TNF-α, further supporting the concept that specific bacterial configurations correspond to distinct local inflammatory and regulatory profiles (Yonezawa et al., 2026). In this context, our associations of Escherichia with IL-6 and MIP-1β and of Phocaeicola with IL-10 fit a broader cervicovaginal pattern in which individual bacterial taxa or community structures are linked to both pro-inflammatory and immunoregulatory mediators, although the specific Phocaeicola–IL-10 relationship appears to be novel and requires independent replication. The viral signal is likewise consistent with Kaelin et al., who showed that cervicovaginal virome composition and bacteriophage–bacterial interactions were associated with genital inflammation and local microbiota structure (Kaelin et al., 2022), supporting cautious interpretation of the Rountreeviridae–MIP-1β association as part of a broader virus–bacteria–immune ecological relationship rather than evidence of direct viral cytokine regulation.

Fungal associations were restricted to the family level. Trichomonascaceae was inversely associated with IL-17A, Eotaxin, EGF, and IFN-γ, whereas Pichiaceae was inversely associated with IL-17E/IL-25, TNF-β, and IL-5. No fungal genus- or species-level associations remained significant after multiple-testing correction, and none of the fungal family-level associations survived global correction across the complete test set. Given the very low fungal classified-read depth and absence of sequenced extraction blanks, these findings should be considered exploratory and should not be interpreted as evidence of direct fungal modulation of the cervical immune environment.

Taken together, the corrected results indicate that microbiome–immune associations were sparse and differed by microbial kingdom and taxonomic resolution. Bacterial associations were restricted to genus-level relationships involving IL-10, IL-6, IL-1RA, MIP-1β, M-CSF, and MIG. Viral associations involved MIP-1β, IL-12(p70), IL-17F, and PDGF-AA, whereas fungal family-level associations involved EGF, Eotaxin, IFN-γ, IL-17A, IL-17E/IL-25, IL-5, and TNF-β. Importantly, only Phocaeicola–IL-10 and Rountreeviridae–MIP-1β remained significant after global correction across the entire multi-kingdom analysis. The predominance and direction of the remaining within-family associations should therefore be interpreted cautiously and in the context of the compositional nature of CLR-transformed abundance data.

The distribution of associations across bacterial, viral, and fungal domains supports evaluating the cervicovaginal ecosystem beyond the bacterial compartment, but it should not be interpreted as evidence that the three microbial domains contribute equally to mucosal immune variation. Differences in sequencing depth, taxonomic resolution, reference-database performance, and microbial biomass vary substantially across kingdoms. In particular, the low viral and fungal read counts increase uncertainty around non-bacterial taxonomic assignments despite the number of within-family FDR-significant associations. Domain-optimized sequencing and targeted validation will therefore be important for determining whether the non-bacterial associations observed here are reproducible in independent cervicovaginal cohorts.

Clinically, these findings reinforce the rationale for considering the cervicovaginal microbiome as a multi-kingdom ecosystem rather than a purely bacterial compartment when evaluating biomarkers of cervical disease risk or candidates for microbiome-directed intervention, such as probiotic restoration of *Lactobacillus* dominance (Schellekens et al., 2025). Differences in the number and taxonomic resolution of significant associations across microbial domains likely reflect both biological variation and substantial differences in sequencing depth and taxonomic detectability. However, the present associations are exploratory and do not establish the clinical utility of multi-kingdom profiles or identify targets for microbiome-directed intervention.

Several limitations should be considered when interpreting these findings. The cross-sectional design precludes causal inference regarding the direction of microbiome-cytokine relationships, and longitudinal sampling will be required to determine whether shifts in taxon abundance precede or follow changes in local cytokine output. Although associations were adjusted for HPV status and cytological grade, residual confounding by unmeasured factors including menstrual cycle phase, sexual behavior, and recent antimicrobial use cannot be excluded. Compared with bacteria, viral and fungal taxonomic measurements were based on substantially lower classified-read depth and therefore carry greater uncertainty despite the presence of several FDR-significant associations. Differences in the number of associations across kingdoms should not be interpreted directly as differences in biological importance because statistical detectability is also influenced by sequencing depth, taxonomic resolution, reference-database coverage, and abundance structure. As in most cervicovaginal microbiome studies, taxonomic assignment at the species level for poorly characterized environmental or low-abundance taxa also carries inherent uncertainty. Furthermore, NILM, ASC-US, LSIL, and ASC-H represent distinct cross-sectional cytological interpretations rather than sequential disease stages within individual participants. In particular, ASC-H is a cytological category indicating that HSIL cannot be excluded and is not equivalent to histologically confirmed HSIL. Although ASC-H specimens showed cytomorphological abnormalities suspicious for high-grade disease, histological confirmation was unavailable. The small number of ASC-H participants (n=9 overall and n=8 in the HPV-positive subgroup) also results in considerable uncertainty around estimates for this category. Furthermore, machine-learning classification analyses remain constrained by sample size relative to the feature dimension (18 cytokine markers). Although cross-validation was implemented to mitigate overfitting, performance estimates carry wide uncertainty intervals. In the absence of an independent external validation cohort, all reported AUC and average precision metrics must be interpreted strictly as internal, exploratory estimates rather than externally validated diagnostic performance. Because of limited specimen volume, multiplex measurements were performed in single wells rather than technical duplicates, precluding sample-specific assessment of replicate variability. Manufacturer-provided assay controls and performance specifications were therefore used for analytical quality assurance.

## 5 Conclusion

This cross-sectional study identified differences in the cervicovaginal immune microenvironment according to HPV status, bacterial community state type, and cervical cytological category. Compositional microbiome reanalysis identified a limited number of bacterial, viral, and fungal associations with cervicovaginal immune mediators after taxonomic curation and multiple-testing correction. Significant bacterial associations were confined to the genus level, fungal associations to the family level, and viral associations were observed across family, genus, and species levels. Of the associations meeting within-kingdom × taxonomic-level FDR criteria, only Phocaeicola–IL-10 and Rountreeviridae–MIP-1β also remained significant after global correction across all tested taxon–immune-marker pairs. Viral and fungal findings should be considered exploratory given the substantially lower non-bacterial sequencing depth and lack of independent taxon-specific validation. These relationships should be considered hypothesis-generating taxon-cytokine associations rather than evidence of direct microbial regulation of the local immune response. Among HPV-positive participants, internally cross-validated cytokine profiles showed moderate discrimination between LSIL and NILM, whereas discrimination of ASC-US was close to chance and estimates for ASC-H were limited by the small sample size. Overall, these findings provide hypothesis-generating evidence of multi-kingdom microbiome–immune associations in HPV-associated cervical abnormalities and support longitudinal and mechanistic studies to determine their temporal and biological significance.

## Data Availability

The datasets presented in this study can be found in online repositories. The names of the repository/repositories and accession number(s) can be found below: https://www.ncbi.nlm.nih.gov/, PRJNA1337009.
