# Supplementary material for: Immune remodeling in HPV-associated cervical disease
Source: Front Cell Infect Microbiol. 2026 Sep 23;16:1918480. doi: 10.3389/fcimb.2026.1918480 (PMC13642807; doi:10.3389/fcimb.2026.1918480)
Supplement: Supplementary file 1 [file Table2.docx]

| **Analyte** | **Control 1 expected range (pg/mL)** | **Control 2 expected range (pg/mL)** |
| --- | --- | --- |
| EGF | 100–207 | 494–1026 |
| Eotaxin | 101–210 | 450–935 |
| G-CSF | 178–370 | 842–1749 |
| GM-CSF | 72–150 | 398–827 |
| IFN-α2 | 201–417 | 1106–2297 |
| IFN-γ | 34–71 | 153–318 |
| IL-1α | 166–345 | 670–1391 |
| IL-1β | 42–87 | 215–446 |
| IL-2 | 17–36 | 85–177 |
| IL-4 | 21–44 | 96–200 |
| IL-6 | 24–43 | 102–212 |
| IL-10 | 84–174 | 402–834 |
| IL-12(p40) | 132–274 | 660–1370 |
| IL-17A | 39–82 | 198–410 |
| IL-17E/IL-25 | 1300–2700 | 6403–13299 |
| IL-18 | 16–33 | 82–170 |
| IL-22 | 434–902 | 2161–4488 |
| IP-10 | 81–169 | 451–936 |
| MCP-1 | 102–212 | 495–1029 |
| M-CSF | 1321–2756 | 6538–13580 |
| MIG/CXCL9 | 150–311 | 764–1586 |
| MIP-1α | 76–158 | 386–801 |
| MIP-1β | 24–49 | 52–107 |
| PDGF-AA | 498–1034 | 2389–4963 |
| PDGF-AB/BB | 309–642 | 1388–2883 |
| TNF-α | 151–313 | 796–1654 |
| TNF-β | 53–111 | 258–537 |
| VEGF-A | 82–171 | 421–875 |
